# Clinical Artificial Intelligence in Non-Small Cell Lung Cancer: From Early Detection and Computational Pathology to Precision Oncology

**DOI:** 10.3390/cancers18213415

**Published:** 2026-10-22

**Authors:** Luca Bertolaccini, Matteo Chiari, Sara Degiovanni, Antonio Mazzella, Giorgio Lo Iacono, Juliana Guarize, Monica Casiraghi, Lorenzo Spaggiari

**Affiliations:** 1 Department of Oncology and Hemato-Oncology, University of Milan, 20122 Milan, Italy; 2 Department of Thoracic Surgery, IEO, European Institute of Oncology IRCCS, 20141 Milan, Italy; 3 Division of Interventional Pulmonology, IEO, European Institute of Oncology IRCCS, 20141 Milan, Italy

**Keywords:** artificial intelligence, lung cancer, digital pathology, radiomics, molecular profiling, precision oncology, multimodal learning

## Abstract

Artificial intelligence (AI) is being developed to analyze radiological, histopathological, molecular, and clinical data across the non-small cell lung cancer (NSCLC) pathway. This narrative review examines representative applications through August 2026, focusing on intended clinical use, validation, and the evidence required to influence care. Studies were selected purposively for clinical relevance and methodological importance. Imaging models support investigation of nodule detection, malignancy estimation, and future lung cancer risk, although predictive performance does not establish the benefit of changing screening or diagnostic pathways. In computational pathology, models classify tissue, quantify morphological features, and estimate selected molecular alterations. Foundation models extend these capabilities, but their evaluation requires attention to pretraining provenance and independence of downstream testing. Histology-based molecular prediction does not currently justify replacing validated molecular assays; prioritization of testing requires prospective workflow evaluation. Models evaluated in immunotherapy-treated cohorts can predict response or survival, but these associations do not establish differential benefit between treatments. The principal limitations include selected retrospective populations, incomplete assessment of calibration and clinically relevant thresholds, uncertain transportability, and limited evaluation of model-guided care. Evidence should therefore be interpreted at the level of the individual system and intended decision. Prospective evaluation, appropriate comparators, explicit failure procedures, and post-deployment monitoring are required before technical performance can be translated into clinical benefit.

## Simple Summary

Artificial intelligence is being investigated across the non-small cell lung cancer pathway, from imaging and tissue assessment to molecular characterization and treatment-response prediction. Its clinical value depends on whether the information it provides improves a specific decision. This review examines representative applications, the studies supporting them, and the limitations that affect their use in practice. Particular attention is given to computational pathology, molecular prediction, and models of immunotherapy response. Accurate classification or prognostic stratification does not by itself establish that a model can replace molecular testing or guide treatment choice. Clinical evaluation should assess errors, effects on diagnostic and treatment pathways, and patient outcomes, with responsibility for decisions remaining clearly assigned to the clinical team.

## 1. Introduction

The contemporary NSCLC pathway is unusually information-dense. A screening or incidental computed tomography (CT) scan may initiate serial imaging, tissue acquisition, histological classification, immunohistochemistry, broad molecular testing, clinical staging, and a multidisciplinary team discussion. Treatment may then depend on resectability, physiological reserve, oncogenic drivers, programmed death ligand 1 (PD-L1) expression, prior exposure to therapy, and the spatial and temporal heterogeneity of disease. No single specialist observes the entire biological process, and no single data source is sufficient for every decision.

**Figure 1:**
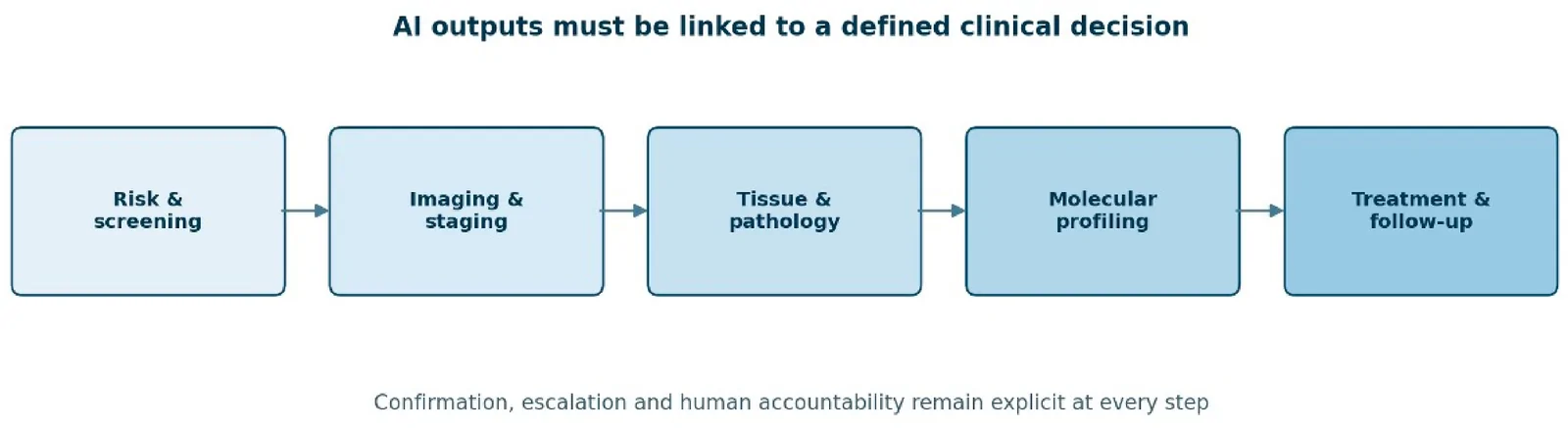
Artificial intelligence across the non-small cell lung cancer (NSCLC) care continuum. Potential applications extend from risk assessment and screening to imaging, tissue acquisition, computational pathology, molecular profiling, treatment selection, and longitudinal monitoring. Each model output should be linked to a defined clinical decision and an explicit confirmation, override, or escalation pathway.

This fragmentation explains much of the interest in AI. Imaging and whole-slide pathology are high-dimensional digital objects; sequencing produces structured molecular measurements; and electronic health records contain repeated observations that could, in principle, be integrated over time. Machine-learning models may detect patterns that are difficult to specify in advance, automate repetitive measurements, and combine a larger number of variables than is practical at the point of care. In NSCLC, proposed uses now extend from screening eligibility and pulmonary nodule detection to histological classification, genotype prediction, prognostication, treatment selection, response assessment and surveillance. These applications and their positions within the NSCLC care continuum are summarized in Figure 1.

The field is nevertheless vulnerable to overstatement. Most models are developed retrospectively in selected datasets, often with case–control sampling, enriched disease prevalence, and incomplete external validation. A technically correct prediction may not answer a clinically useful question. A model that reduces reporting time may fail to shorten the diagnostic pathway; a slide-based mutation prediction may appear accurate yet remain unsafe as a replacement for sequencing; and a prognostic model may stratify risk without identifying which treatment changes that risk. These distinctions are essential because discrimination, clinical utility, and causal treatment benefit are not interchangeable.

This review examines how evidence supporting AI applications relates to the decisions they are intended to inform across the NSCLC pathway. Rather than organizing the discussion solely by technology, it considers when the required inputs become available, what action could follow the output, and whether the study design supports that action. Particular attention is given to computational pathology, molecular inference, and treatment-response prediction. The purpose is to distinguish technical performance, clinical validity, and demonstrated benefit from model-guided care.

## 2. Literature Identification and Scope

This state-of-the-art narrative review was informed by PubMed/MEDLINE searches conducted from database inception to 31 August 2026, limited to English-language publications. The search combined lung cancer and NSCLC terms with terms for artificial intelligence, machine learning, imaging, computational pathology, molecular profiling, treatment response, prognosis, and multimodal models. The search strategy and selection criteria are provided in Supplementary Tables S1–S3.

Study selection was purposive rather than exhaustive. Priority was given to studies addressing a defined clinical use, with particular attention to independent validation, prospective workflow evaluation, relevant comparators, and clinically interpretable outcomes. Seminal development studies were retained where needed to explain an application. Reference lists provided additional sources.

Screening, pulmonary nodule assessment, and tissue-acquisition studies were included because they concern the pathway before histological confirmation. Their findings were not treated as specific to NSCLC unless supported by the study population. Studies of mixed cancer populations and general-purpose pathology models were used to inform methodological discussion, without assuming equivalent performance in NSCLC.

For the principal studies, the synthesis distinguishes publication status, study design, intended use, population, model inputs, validation setting, comparator, reported results, and limitations relevant to clinical interpretation. Contextual studies of biopsy strategies or molecular assays are identified separately from studies directly evaluating AI. The selected studies are summarized in Table 1. Study-specific methodological considerations and their implications for interpretation are detailed in Supplementary Table S5.

No new effect estimates or pooled analyses were calculated. The review addresses different clinical tasks, populations, inputs, and outcomes for which a common summary estimate would not answer a coherent clinical question. Reliance primarily on PubMed/MEDLINE and English-language publications may have underrepresented the technical literature and studies indexed elsewhere. Supplementary citation searching did not ensure comprehensive coverage.

Records were screened by title and abstract, and full texts were retrieved for all studies considered potentially relevant. Literature identification and initial selection were performed by MC and SD. Study characteristics and numerical findings were extracted by MC and SD and checked against the original reports by LB. Priority was given, in descending order, to: (i) randomized or prospective evaluations embedded in a clinical workflow; (ii) studies with independent external, temporal, geographic, or multicenter validation; (iii) studies comparing a model with clinicians, validated clinical scores, or standard-of-care biomarkers; (iv) studies reporting calibration, threshold-specific performance, predictive values, workflow effects, or patient outcomes rather than discrimination alone; and (v) earlier seminal studies that established an application or a methodological standard. Single-institution retrospective studies without external validation were cited only when they illustrated a recurring methodological problem or an important proof of concept. For each included study, the following were extracted and used to weigh the evidence: intended clinical use and the decision the model was meant to change; data provenance (institutions, scanners, staining and preparation protocols); patient-level separation of training and evaluation data; reference standard and adequacy of follow-up for benign or negative results; comparator; discrimination, calibration, and threshold-specific metrics; subgroup and out-of-distribution performance; and the handling of missing data and model failure. Because this was a narrative rather than a systematic review, no pooled effect estimate was calculated, and no formal risk-of-bias score was assigned to individual studies; PROBAST-informed reasoning was instead applied narratively to the evidence base of each clinical task.

AI is used here as an umbrella term. Machine learning refers to models that learn relationships from data rather than relying solely on hand-coded rules. Deep learning refers to multilayer neural networks, including convolutional architectures, vision transformers, and multimodal foundation models. Radiomics conventionally denotes the extraction of predefined quantitative imaging features, whereas deep radiomics learns representations directly from images. Pathomics applies related approaches to digitized tissue. (Operational definitions are provided in Supplementary Table S4.) These categories overlap, and their labels are less important than the model’s intended use, input data, reference standard, and consequences of error. The literature search was limited to English-language publications and was not designed to identify every technical proof-of-concept study. The review therefore emphasizes clinical relevance and validation rather than exhaustive coverage.

Study limitations were considered qualitatively with reference to participants and data sources, predictors, outcomes, and analysis, informed by PROBAST + AI [11]. Particular attention was given to separation of development and evaluation data, availability of inputs at the intended decision point, outcome ascertainment, and applicability to the proposed population. This was not a formal PROBAST + AI assessment, and no study-level scores or overall risk-of-bias ratings were assigned. Incomplete reporting was distinguished from a documented methodological limitation.

## 3. Screening, Early Detection and Pulmonary Nodule Assessment

Low-dose CT reduces lung cancer mortality in appropriately selected high-risk populations. Still, population-scale screening imposes a substantial interpretive burden and detects many nodules that will never become clinically important [12,13]. AI has therefore been developed for three related but distinct tasks: identifying nodules, determining whether a detected nodule is malignant, and predicting future lung cancer risk from the entire scan.

Nodule-detection systems can serve as concurrent or second readers and may improve sensitivity, particularly for small or easily overlooked lesions. Their practical value depends on the false-positive burden, the handling of perifissural nodules, scars and vascular structures, and whether the software is used in a screening cohort or an unselected clinical population. Performance in curated thin-section CT datasets cannot be assumed to persist in routine examinations reconstructed with different kernels, slice thicknesses, and scanners. In a retrospective evaluation of 102 routine 5-mm-section CT examinations containing 353 nodules or masses, radiologist sensitivity ranged from 90.4% to 94.3%, compared with 80.2% to 89.2% for the two commercial systems. Radiologists also generated fewer false-positive findings per scan (1.61–3.56 vs. 5.28–7.97) [14]. These results do not establish general human superiority, but they show that performance on curated thin-section datasets may deteriorate in heterogeneous routine imaging.

Malignancy prediction is a different task. Deep-learning models can use nodule morphology, the surrounding lung, and longitudinal change, whereas established clinical models use variables such as age, smoking history, nodule size, location, spiculation, and emphysema. The appropriate comparison is therefore not AI against unaided intuition, but AI-assisted assessment against validated clinical-radiological models and guideline-based management. Threshold selection matters more than a small difference in area under the receiver operating characteristic curve (AUROC): a false-negative result may delay diagnosis, whereas a false-positive result can lead to repeated CT, positron emission tomography, biopsy, or surgery.

Whole-scan risk models further broaden the question. Ardila et al. developed an end-to-end three-dimensional deep learning system using screening CT examinations and demonstrated performance comparable to radiologists in an observer study [15]. Sybil subsequently demonstrated that future lung-cancer risk over one to six years could be estimated from a single low-dose CT without requiring demographic or smoking variables [1]. External evaluations are important because long-horizon risk prediction is sensitive to cancer incidence, eligibility criteria, competing risks, and the prevalence of granulomatous or subsolid disease. A large Asian cohort reported good short-term performance, including among never-smokers, but also showed lower performance in some radiological subgroups and attenuation over longer prediction horizons [16]. A 2025 validation in a racially diverse United States screening program—2092 baseline examinations, 62% of participants identifying as non-Hispanic Black—found broadly preserved one- to six-year discrimination across racial and socioeconomic subgroups [17], a result that partially answers the transportability concern while still coming from a single urban program. Together, these findings justify continued study of personalized screening intervals; they do not yet justify replacing established eligibility and follow-up policies.

The endpoint used for implementation should reflect the proposed benefit. Workload reduction requires a high-sensitivity rule-out strategy and a safety mechanism for discordant cases. Earlier diagnosis requires evidence on stage distribution and time to definitive management, not simply faster image reporting. LungIMPACT tested chest-radiograph prioritization rather than AI availability: AI was available in both groups, with prioritization randomized by day. Reporting was faster, but the trial did not demonstrate a significant reduction in time to CT or lung cancer diagnosis. These findings apply to the intervention and pathway evaluated; they do not establish equivalence or exclude benefit from other AI-supported workflows. The distinction between a faster intermediate process and an improved diagnostic pathway is therefore clinically important [2].

## 4. Imaging Phenotypes, Staging and Longitudinal Assessment

Radiomics was among the earliest attempts to convert tumor images into quantitative biomarkers. Texture, shape, intensity and wavelet-derived features have been associated with histology, genotype, survival and treatment response [18]. Deep learning reduces dependence on hand-crafted features and may capture peritumoural or whole-lung information, but it does not remove the need for standardized acquisition, segmentation and validation.

Imaging phenotypes can contribute to histological classification and the estimation of invasive components in subsolid nodules. They may also support assessment of nodal or distant disease where size-based rules are imperfect. Yet pathology and staging labels often contain noise. If clinical stage rather than systematic pathological nodal assessment is used as the reference standard, the model may reproduce an incomplete work-up. Similarly, a model trained to predict histology from CT may learn differences in acquisition or referral patterns that correlate with histology rather than with tumor biology.

Longitudinal imaging is clinically more informative than a single scan but methodologically more challenging. Serial examinations differ in breath-hold, positioning, reconstruction, and treatment-related inflammation. Registration errors can be mistaken for biological change. Conventional Response Evaluation Criteria in Solid Tumors remain limited for immunotherapy, in which pseudoprogression, immune-related inflammation, and heterogeneous lesion-level responses can occur. Quantitative models have been associated with response and survival after immunotherapy or radiotherapy [19,20,21]. The serial-CT study included separate discovery, routine-clinical-practice tests, and external trial cohorts, as detailed in Table 1. Because the score uses baseline and approximately 12-week CT information, it is an on-treatment prognostic assessment rather than a pretreatment selection tool. Its interpretation depends on the availability of follow-up imaging and the survival-analysis time origin. Patients who deteriorate or die before reassessment may not be represented by the intended application. Improved survival discrimination does not establish that changing treatment according to the score improves outcomes [3].

Imaging-based molecular prediction is a proposed approach to testing prioritization or discordance detection. Its use requires evidence that the resulting workflow does not delay or prevent indicated molecular testing. A negative radiomic prediction cannot be interpreted as exclusion of an actionable alteration.

## 5. AI-Assisted Bronchoscopy and Tissue Acquisition

The expansion of CT screening and the increasing detection of incidental nodules are enlarging the population of patients with small peripheral pulmonary lesions requiring diagnostic clarification. AI could assist airway segmentation, route planning, tool-in-lesion confirmation, and prediction of diagnostic yield.

The clinically meaningful target is not navigation success alone. A catheter can reach the planned coordinates, yet the biopsy remains non-diagnostic due to CT-to-body divergence, respiratory motion, eccentric radial ultrasound position, necrosis, or inadequate sampling. VERITAS provides contextual evidence on biopsy strategies and patient-level diagnostic outcomes, not a direct estimate of AI benefit. Navigational bronchoscopy was noninferior to transthoracic needle biopsy for diagnostic accuracy and was associated with fewer pneumothoraces. The trial did not isolate the contribution of a learned algorithm. Its results should not be directly compared with yields from unrelated robotic bronchoscopy series without considering differences in populations, platforms, definitions, and follow-up [9]. Models should therefore be trained and evaluated against patient-level diagnostic yield, with adequate follow-up of benign results, rather than against a procedural surrogate. They should also account for lesion size, bronchus sign, density, lobe, distance from pleura, confirmation modality, and operator learning. Navigation success, tool-in-lesion confirmation, and diagnostic yield should therefore be reported separately, because they represent different stages of procedural performance.

AI may also contribute to tissue stewardship. When only a small sample is available, real-time estimates of adequacy or tumor cellularity could help allocate material between cytology, immunohistochemistry and next-generation sequencing. This is especially relevant when repeated biopsy carries risk. However, any system influencing specimen allocation must be validated on the actual preparation types used in practice, including smears, cell blocks, frozen sections and small forceps or needle biopsies. Evidence for AI-assisted adequacy assessment during bronchoscopy remains preliminary. This application should therefore be presented as a development priority rather than an established clinical capability.

## 6. Computational Pathology: From Classification to Quantitative Phenotyping

Whole-slide imaging has made routine histology accessible to modern computer vision. Early work showed that deep neural networks could distinguish lung adenocarcinoma, squamous cell carcinoma, and non-neoplastic tissue, and predict selected mutations from hematoxylin- and eosin-stained slides [22]. Weakly supervised learning subsequently reduced the need for exhaustive pixel-level annotations by training on slide-level labels [23]. These approaches align well with routine pathology, where diagnostic labels are abundant but manual delineation is costly.

Defined tasks such as tumor detection and quantitative measurement allow direct assessment of model errors and specialist review of the output. Algorithms can identify tumor regions, support histological classification, count mitoses, quantify immune cell density, and measure biomarker expression. Such tools may improve consistency and release pathologist time, particularly when the output is visually reviewable. Their error profile, however, is shaped by tissue processing, scanner characteristics, staining, section thickness, artifacts, and the case mix used for training. External validation must therefore be performed at the laboratory and scanner level, with predefined rules for poor-quality or out-of-distribution slides.

Slide-level subtype classification is not equivalent to a complete pathological diagnosis. NSCLC diagnosis incorporates growth pattern, cytological features, immunohistochemistry and clinical-radiological context. Rare variants, mixed tumors, mucinous differentiation, and small, crushed biopsies may be underrepresented in training sets. Models that perform well on resection specimens may fail on cytology or scant biopsies, which are precisely the specimens in which diagnostic assistance might be most attractive.

Computational pathology can also quantify spatial organization. The location and density of tumor-infiltrating lymphocytes, tumor–stroma relations, tertiary lymphoid structures and intratumoural heterogeneity may contain prognostic or predictive information not captured by routine categorical reporting. Pathomic features have been associated with survival and molecular phenotypes [24,25]. The principal difficulty is reproducibility across tissue sampling, staining, scanning, and analytical pipelines. Many spatial features are sensitive to tissue area, sampling, stain normalization, and the definition of cell phenotypes. Before adoption, an interpretable measurement should be shown to add information beyond stage, histology, established biomarkers, and basic image-quality variables.

Foundation models trained via self-supervision on very large collections of slides may improve data efficiency and enable adaptation to multiple downstream tasks [26,27,28]. The newest generation extends this idea to slide-level representations: Prov-GigaPath was pretrained on more than 1.3 billion tiles from 171,189 real-world slides [29], and TITAN combines whole-slide representations with language-based information to support several downstream pathology tasks [30]. Their scale is appealing, particularly for rare labels, but it creates new validation problems. Pretraining datasets may overlap with evaluation cohorts, provenance may be incompletely reported, and apparent generality may conceal weak performance in under-represented tumor types or laboratories. An independent benchmark evaluated 19 pathology foundation models across 13 cohorts and 31 downstream tasks. The study addressed independence of evaluation data, including the use of selected CPTAC collections without TCGA patient overlap. Its findings support task-specific model comparison, rather than the assumption that a high-performing foundation model constitutes a clinically validated NSCLC system [4].

## 7. Inferring Molecular Alterations from Routine Histology

The possibility of reading genotype from morphology is one of the most compelling applications of AI in NSCLC. Histology reflects the downstream consequences of oncogenic signaling, differentiation, and interactions with the tumor microenvironment. Deep learning models have therefore been trained to predict EGFR, KRAS, STK11, TP53, and other alterations from routine slides [22,31]. Similar strategies have been used to estimate gene-expression programs, immune phenotypes, and PD-L1 status.

The intended use must be defined before performance is interpreted. A prescreening model could enrich samples for urgent molecular analysis or identify cases for repeat testing when tissue is scarce. A quality-control model could flag discordance between morphology and a laboratory result. A replacement test would require a substantially higher evidentiary threshold because false-negative predictions could deny effective targeted therapy. A 2025 systematic review included 16 studies of EGFR mutation prediction from whole-slide images, but only four contributed to the meta-analysis. The reported estimates are summarized in Table 1. They do not establish a safe operating threshold for withholding molecular testing, particularly across populations with different mutation prevalence and specimen characteristics [5]. For most actionable alterations, current slide-based models lack the sensitivity, calibration, multicentre robustness, or prospective evidence required for exclusionary use. The acceptable threshold depends on the proposed action. A model used only to prioritize samples for sequencing can tolerate lower specificity if sensitivity remains high. By contrast, a model used to withhold molecular testing would require an exceptionally low and prospectively validated false-negative rate for every actionable alteration. Aggregate AUROC across several genes cannot establish safety for this purpose.

Class-specific performance should be reported separately for each alteration, because a high macro-averaged AUROC may conceal clinically unacceptable sensitivity for a rare but actionable target. Prevalence is central. Positive and negative predictive values vary across populations, histological subtypes, smoking distributions, and disease stages, even when sensitivity and specificity remain stable. Case–control development datasets often distort this relationship. Reporting AUROC alone is insufficient; clinically meaningful thresholds, predictive values, calibration, and the number of molecular tests saved or missed should be provided at the target prevalence.

Tumor heterogeneity adds another limitation. A resection section samples a different volume and disease time point from a small diagnostic biopsy or a progressing metastatic lesion. Molecular evolution under treatment can render the primary tumor an imperfect proxy for resistance mechanisms. AI inference from baseline morphology is therefore unlikely to replace direct analysis when the clinical question concerns an acquired alteration. Testing prioritization and quality-control support remain proposed uses requiring workflow validation. Binary mutation prediction is also distinct from identification of the specific actionable variant. Neither should be used to justify omission of indicated molecular testing without evidence supporting that decision.

## 8. AI in Liquid Biopsy, Molecular Profiling and Decision Support

Machine learning is also used downstream of molecular assays. In circulating tumor DNA, algorithms contribute to error suppression, fragmentomic analysis, methylation signatures and the combination of weak signals across genomic regions. These methods may support early detection, assessment of minimal residual disease, and monitoring of resistance. Their performance is strongly stage-dependent because tumor shedding is lower in small, indolent, or anatomically confined disease. Models developed in symptomatic or advanced cancer should not be extrapolated to screening or postoperative surveillance without stage-specific validation, because circulating tumor DNA abundance and detection sensitivity differ substantially across these settings. Minimal residual disease (MRD) illustrates both the promise and the difficulty. LEMA and LUCID evaluated recurrence prediction using post-treatment ctDNA sampling. The sensitivity and specificity summarized in Table 1 refer to longitudinal detection, not a single postoperative landmark test. The reported negative predictive value of 88% concerned patients with stage II–III disease who were ctDNA-positive before treatment and negative afterward. These findings describe recurrence-risk stratification; they do not establish the benefit of treatment escalation or omission based on the assay. This study is included as contextual evidence on molecular monitoring and does not quantify the incremental contribution of AI [10]. A prognostic association of this kind does not, by itself, establish that acting on the test improves outcomes. MERMAID-1 (NCT04385368/EudraCT 2020-000556-35) evaluated adjuvant durvalumab with chemotherapy against placebo with chemotherapy after complete resection. MERMAID-2 (NCT04642469/EudraCT 2020-000612-30) evaluated durvalumab against placebo in patients with MRD after surgery and curative-intent therapy. Neither should be described as ongoing: public results records document study end dates in 2023 and 2024, respectively. MERMAID-2 enrolled 30 participants and ended early, with descriptive analyses. These studies do not establish the clinical utility of an AI-guided MRD treatment strategy [32]. AI-enhanced assays require analytical validation, clinical validation, and—when used to allocate therapy—evidence of clinical utility. Lead-time gains should not be confused with survival gains, and surveillance strategies must reckon with false positives, clonal hematopoiesis, and the psychological weight of molecular relapse without an established intervention.

For broad sequencing panels, AI can assist variant annotation and prioritization, infer functional effects and link complex molecular profiles to clinical trials or therapies. Large language models may help summarise reports and retrieve evidence, but they introduce a separate risk: plausible language can obscure unsupported or outdated recommendations. Any generated recommendation should display the underlying variant, assay limitations, evidence level, source, and retrieval date. Unsupported text generation must be treated as a system failure rather than as an acceptable limitation of the interface.

## 9. Prognosis, Treatment-Response Prediction, and Implications for Treatment Selection

Numerous AI models predict recurrence, progression, or survival using imaging, pathology, molecular data, or electronic records. Prognostic modeling is useful for counseling, surveillance design, and trial stratification, but only when it adds information beyond readily available clinical variables and is well calibrated in the target setting. Deep-learning survival models based on CT have shown that imaging contains prognostic information beyond conventional staging [33]. Pathology models likewise capture tumor and microenvironmental features associated with outcome. The treatment discussion focuses on prognosis and response prediction, particularly in immunotherapy-treated populations. It is not an exhaustive assessment of surgical planning, radiotherapy delivery, or drug development. These tasks require different comparators and endpoints and should not be inferred from the performance of an immunotherapy-response model.

Treatment selection requires evidence about outcomes under clinically relevant alternatives. A model evaluated only in patients receiving a particular treatment may predict response or prognosis without identifying which treatment would be preferable. Deep-IO was evaluated in patients receiving ICI monotherapy. External discrimination for objective response was similar to that of PD-L1, and combining the two scores improved discrimination in the reported cohort (Table 1). This does not demonstrate formal equivalence or differential benefit from ICI compared with another treatment. Path-IO remains conference-reported evidence. Its external evaluation included Lung-MAP S1400I, which compared nivolumab with nivolumab plus ipilimumab. That comparison cannot substantiate a claim about choosing ICI monotherapy over chemoimmunotherapy. The available abstract does not provide the interaction estimate and uncertainty needed to support a treatment-by-score interaction supporting treatment selection. Biological plausibility does not resolve this distinction. Treatment-selection claims require an appropriate comparative design and subsequent evaluation of the outcomes of model-guided care [6,7]. Studies using radiomics to predict immunotherapy response have reported promising discrimination [19,20,21], and multimodal integration of radiology, pathology, and clinical variables has improved prediction in retrospective NSCLC cohorts [7]. None of these models has yet been tested prospectively against patient outcomes, and treatment-response datasets remain small, selected, and confounded by line of therapy, performance status, mutation profile, and imaging schedule.

The distinction between predictive and prognostic modeling should be explicit in study design. Treatment-by-biomarker interaction should be prespecified and evaluated directly, preferably in randomized-trial data; separate demonstrations of statistical significance within treatment subgroups are not sufficient evidence of interaction. When only observational data are available, causal assumptions, treatment assignment, immortal time bias, and informative censoring require careful handling. Comparators should match the intended decision and treatment setting. Established treatment-selection biomarkers, exploratory biomarkers, and clinical prognostic factors should not be treated as interchangeable benchmarks.

Applicability should be considered separately in resectable disease, unresectable stage III disease, and metastatic disease. In resectable NSCLC, prediction of occult nodal disease, recurrence, or pathological response does not establish which operation or perioperative regimen improves outcome. In unresectable stage III disease, models of prognosis, response, and toxicity address different decisions from those involved in radiotherapy planning or treatment allocation. In metastatic disease, interpretation depends on treatment line, regimen, driver status, PD-L1 expression, performance status, and specimen timing. Results from an ICI-treated cohort should not be extended to oncogene-driven disease or targeted-therapy selection without relevant validation.

## 10. Multimodal AI and the NSCLC Digital Phenotype

Multimodal models combine information that may be complementary but is not always available in the same patients or at the same clinical time point. Vanguri et al. evaluated a model integrating radiological, pathological, and genomic features in 247 patients with advanced NSCLC. The reported multimodal performance was obtained through internal cross-validation; additional radiology and pathology cohorts evaluated individual modalities rather than independently validating the complete multimodal model. This distinction matters when interpreting the improvement over single-modality measures. For conference-reported systems such as Path-IO, the available description also limits assessment of incremental performance and transportability [7] and the Path-IO experience suggests that adding radiomics and clinical variables to pathology-based scores raises discrimination further [8]. Emerging vision-language and foundation-model architectures may further connect slide regions, images, reports, and molecular labels. Multimodal integration may occur at the input level, through joint learned representations, or by combining predictions from modality-specific models. These approaches make different assumptions about missingness and temporal alignment and should not be treated as analytically equivalent.

Adding modalities increases the available information but does not necessarily improve transportable clinical performance. Missingness is rarely random: patients with sufficient tissue receive broader testing, fitter patients undergo more complete staging, and progression prompts additional imaging. A multimodal model may learn these care patterns instead of tumor biology. Requiring every modality can also reduce eligibility and worsen inequity. Models should specify how they operate when an input is absent, whether performance is preserved with partial data, and whether a simpler model achieves similar clinical utility.

Temporal alignment is equally important. A baseline biopsy, a CT obtained after therapy, and a molecular result from a later resistant lesion do not reflect the same biological state. Data fusion should preserve time and specimen provenance rather than treating all patient-level information as contemporaneous. External validation should reproduce the intended sequence of availability: a model used before biopsy cannot include information generated after pathology, even indirectly.

Large language models may eventually provide an interface for multimodal decision support, summarising the record and presenting evidence to the thoracic oncology multidisciplinary team. Their fluency is not reliability. Retrieval, citation fidelity, version control and abstention are essential, and recommendations must remain reviewable by the clinicians responsible for the decision. The appropriate endpoint is whether the system improves decision quality, consistency, timeliness, or trial access without increasing the rate of unsafe recommendations.

## 11. Why High Model Performance Often Fails to Translate

### 11.1. Dataset Construction and Leakage

Data leakage can occur at the patient, slide, lesion, or time level. Tiles from the same slide, multiple nodules from the same patient, or serial examinations must not be divided across training and test sets. Preprocessing and feature selection must be fitted within the training data. When public datasets are repeatedly used for development and evaluation, informal overfitting to the benchmark can occur even without direct leakage. When cross-validation is used, all observations derived from the same patient must remain within the same fold. A separate test set is not independent if its results were used to inform preprocessing choices, model selection, or threshold tuning.

### 11.2. Spectrum, Selection and Reference-Standard Bias

Diagnostic case–control datasets often include unequivocal cancer cases and healthy controls rather than the indeterminate cases encountered in practice. Histology cohorts over-represent resected tumors and under-represent patients without tissue confirmation. Molecular models may exclude failed assays, although assay failure is part of the intended workflow. Reference standards should reflect the clinical question and include adequate follow-up for presumed benign disease.

### 11.3. Generalisability and Hidden Shortcuts

Models can exploit scanner, staining, compression, hospital, or acquisition signatures that correlate with outcomes in the development data. A system may appear to recognize tumor biology while identifying the institution from which the image originated. Temporal and geographically external validation, scanner- and laboratory-subgroup analyses, and stress testing after plausible acquisition changes are therefore necessary.

### 11.4. Discrimination Without Calibration or Utility

AUROC measures ranking across thresholds; it does not indicate whether predicted probabilities are accurate or whether using the model improves decisions. Calibration plots, calibration-in-the-large, calibration slope, clinically relevant sensitivity and specificity, predictive values, decision-curve analysis and net benefit should be reported as appropriate. Thresholds should be chosen from the development data and evaluated without change in external cohorts.

### 11.5. Inappropriate Human Comparison

Claims that AI equals or outperforms clinicians are often based on small reader studies without representative workflow, prior imaging, or clinical information. The clinically relevant comparison is usually between clinicians who use AI and those who do not. Evaluation should include reading time, automation bias, disagreement resolution, and performance across experience levels. Human–AI interaction can improve or worsen performance depending on how confidence and explanations are presented. Reader studies should report the number and experience of readers, the case-selection procedure, the washout period, the reading order, the clinical information available, and the statistical treatment of reader and case variability.

### 11.6. Evidence Standards for Clinical Translation

The required evidence depends on risk and intended use. A low-risk measurement aid, reviewed by a pathologist, does not require the same level of evidence as an autonomous system that excludes molecular testing. Nonetheless, several principles apply across applications. The dataset should represent the target population and workflow; the comparator should be the current standard of care; the evaluation should be external and preferably prospective; and errors should be analyzed by clinically relevant subgroups. Evidence requirements according to intended use are summarized in Table 2.

PROBAST + AI addresses the quality, risk of bias, and applicability of prediction-model development and evaluation. TRIPOD + AI addresses reporting of prediction models, while CONSORT-AI, SPIRIT-AI, and DECIDE-AI concern distinct stages of clinical evaluation. Adherence to a reporting guideline does not itself establish low risk of bias or clinical utility [34,35,36,37,38]. These frameworks should be used prospectively as design tools rather than applied cosmetically after analysis.

**Figure 2:**
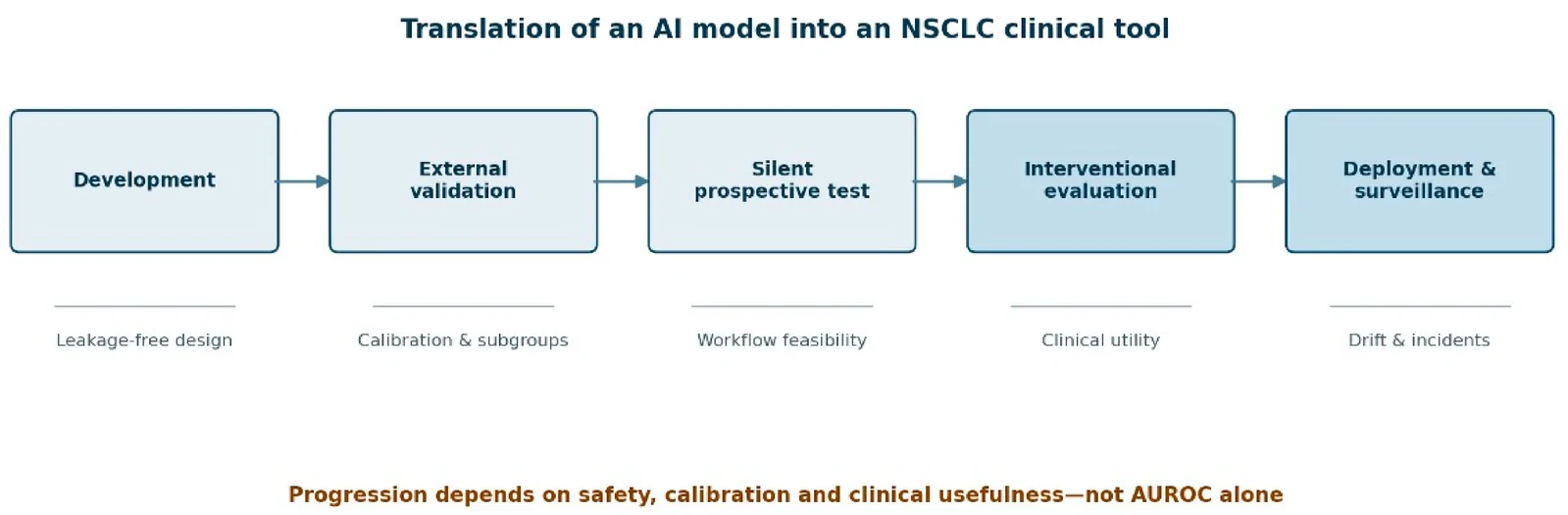
Translational pathway for clinical artificial intelligence in non-small cell lung cancer. Development and internal validation should be followed by external validation across geographic and temporal settings, prospective silent evaluation, interventional comparison with standard care, and post-deployment surveillance. Progression between stages should depend on calibration, subgroup safety, workflow performance, and clinical utility rather than discrimination alone.

Prospective silent evaluation is a useful intermediate step. It tests data availability, latency, missingness, drift, and calibration without influencing care. Interventional studies should follow, using endpoints matched to the proposed mechanism. A triage tool may be judged by turnaround time and missed urgent cases; a nodule classifier by invasive procedures avoided without delayed cancer detection; a molecular prioritization tool by testing completion, turnaround time, and actionable alterations delayed or missed; and a treatment-selection model by patient outcomes, not predicted response alone. A staged translational pathway from development to post-deployment surveillance is shown in Figure 2.

Randomization may occur at patient, clinician, session, or center level. Cluster designs can reduce contamination when clinicians cannot realistically ignore previous exposure to the system. Stepped-wedge designs may be practical for service implementation but require attention to temporal trends. Whatever the design, failure handling and clinician override should be predefined, and analysis should follow the assigned intervention rather than only successful model outputs.

## 12. Equity, Governance and Post-Deployment Surveillance

NSCLC incidence, histology, driver prevalence, smoking exposure and access to molecular testing vary across sex, ancestry, geography and socioeconomic position. Models trained in heavily screened or surgically treated populations may not serve patients diagnosed through emergency or symptomatic routes. Subgroup performance should therefore be evaluated at clinically meaningful thresholds, with uncertainty intervals and intersectional analyses where sample size permits. A globally accurate model can still systematically underperform in a smaller group. The consistent performance of the Sybil risk model in a predominantly Black screening cohort is encouraging [17], but single-program validation cannot substitute for surveillance across the populations in which a model is actually deployed.

Governance begins with a defined clinical question and named accountability. The model’s input requirements, output, intended user, contraindications, and action thresholds should be documented. Clinicians need to know when the system is likely to fail and how to proceed when its recommendation conflicts with other evidence. Patients should receive proportionate information when AI materially influences diagnosis, prognosis, or treatment recommendations, particularly when the system changes the available clinical options.

Regulatory requirements depend on the system’s intended purpose, applicable product legislation, risk classification, and implementation timetable. AI systems used in healthcare should not be assumed to fall under a single regulatory category. Regulatory conformity and clinical utility are separate considerations: authorization does not establish benefit for every population or workflow in which a system might be used [39]. Whether a particular system is regulated as high risk depends on its intended purpose and, where applicable, its status under medical-device legislation. Institutions therefore retain responsibility for procurement, validation, training, cybersecurity, incident reporting and ongoing audit.

Post-deployment surveillance should monitor input drift, missingness, calibration, subgroup performance, overrides and clinically significant errors. Updating a model can alter its risk profile and should trigger version control and revalidation. Automation bias deserves explicit attention: a plausible but incorrect output may be more dangerous than no output, especially when workload is high. Conversely, repeated false alarms lead to alert fatigue and abandonment.

## 13. A Practical Translational Framework

The path from a promising model to a useful NSCLC tool can be organized around five questions. First, what decision will change? Second, at what point in the pathway are the required data genuinely available? Third, what error is clinically unacceptable? Fourth, against which existing workflow should the system be compared? Fifth, how will performance be monitored after deployment?

This framing prevents a common reversal in AI research, in which an available dataset determines the question. It also encourages parsimonious models. If a transparent clinical model performs as well as a complex network, the simpler approach may be preferable. If AI contributes mainly by automating a measurement, it should be evaluated as an automation tool rather than described as precision oncology. Representative applications, proposed benefits, meaningful endpoints, and principal failure modes are summarized in Table 3.

For computational pathology and molecular profiling, a tiered implementation model is more realistic than immediate diagnostic substitution. AI may first support quality control, tumor detection, and quantitative measurement. It may then triage cases for additional stains or molecular testing. Only after robust prospective evidence should it influence exclusionary or therapeutic decisions. Multimodal decision support belongs at the final tier because its apparent comprehensiveness creates both greater opportunity and greater potential for concealed error.

## 14. Future Directions

The next phase of NSCLC AI should be judged less by incremental gains in retrospective AUROC and more by performance in clinically embedded evaluations. Several developments are promising. Self-supervised foundation models may reduce annotation requirements; federated or distributed approaches may enable multicentre learning without centralizing identifiable data; and multimodal models may link tumor morphology to imaging, genomics, and clinical course. Prospective registries can provide continuous evaluation across changing technologies.

Methodological priorities are equally important. Models should be compared against strong baselines, validated over time and across geographies, and tested under acquisition shifts. Uncertainty and abstention should be treated as core functions. Treatment models should address causal questions rather than relabel prognostic association as therapeutic prediction. Economic evaluation should include downstream tests, procedures, staffing, and error consequences, rather than software cost alone.

The role of the multidisciplinary team will persist. AI can organize information, expose discordance, and quantify patterns. However, NSCLC decisions also depend on patient preferences, frailty, technical resectability, local expertise, and values that are not fully represented in datasets. The most useful system may therefore be one that makes its evidence and uncertainty visible, prompts the right discussion, and records why a recommendation was accepted or rejected.

## 15. Conclusions

AI studies in NSCLC demonstrate technical capabilities across imaging, computational pathology, molecular inference, and outcome prediction. Their clinical implications depend on the individual system, population, and intended decision. External validation improves confidence in performance but does not establish that using the output benefits patients.

Molecular prediction should not be treated as equivalent to validated testing, and response prediction within a treated cohort does not establish differential treatment benefit. Multimodal systems require additional scrutiny of data availability, timing, and independence of evaluation.

Further evaluation should measure the consequences of model-guided care, including diagnostic errors, testing completion, unnecessary procedures, treatment allocation, and patient outcomes. Adoption should follow evidence for the proposed use, with defined failure procedures, clinician responsibility, and ongoing performance monitoring.

## Figures and Tables

**Table 1:** Selected evidence informing the clinical interpretation of AI applications across the lung cancer and NSCLC pathway. Studies were selected to illustrate the evidence supporting the principal claims discussed in the review; the table is not an exhaustive inventory. Estimates are reproduced from the cited reports and were not recalculated. Evidence-stage descriptions refer to the evaluation performed, not regulatory authorization or routine adoption. Where applicable, examinations, slides, and patients are distinguished. AI, artificial intelligence; AUROC, area under the receiver operating characteristic curve; CGMH, Chang Gung Memorial Hospital; CI, confidence interval; CT, computed tomography; ctDNA, circulating tumor DNA; H&E, hematoxylin and eosin; HR, hazard ratio; ICI, immune checkpoint inhibitor; LDCT, low-dose computed tomography; MGH, Massachusetts General Hospital; NLST, National Lung Screening Trial; NSCLC, non-small cell lung cancer; OS, overall survival; PD-L1, programmed death ligand 1; PFS, progression-free survival; RECIST, Response Evaluation Criteria in Solid Tumors.

Study; Publication Status	Clinical Task and Population/Input	Design and Sample	Validation and Comparator	Principal Reported Finding	Main Limitation and Clinical Implication
[1]; full article	Future lung cancer risk from one LDCT examination; screening populations	Retrospective development using NLST; evaluation on 6282 NLST, 8821 MGH, and 12,280 CGMH examinations	Held-out NLST evaluation; external MGH and CGMH cohorts	One-year AUROC: 0.92 (95% CI, 0.88–0.95), 0.86 (0.82–0.90), and 0.94 (0.91–1.00), respectively	Examination counts are not patient counts. Risk discrimination does not establish benefit from changing eligibility or screening intervals. Evidence stage: external validation.
[2]; full article	Prioritization of primary-care chest radiographs within the diagnostic pathway	Prospective multicenter trial; 93,326 radiographs; prioritization randomized by day	Prioritization on versus off; AI available in both groups	Median reporting time: 34.1 versus 47.0 h. Time to CT: geometric-mean ratio 0.97 (95% CI, 0.93–1.02); time to diagnosis: 0.98 (0.83–1.16)	Faster reporting did not demonstrate faster downstream diagnosis. Results concern prioritization in this pathway, not all diagnostic AI. Evidence stage: comparative workflow evaluation.
[3]; full article	On-treatment survival assessment in advanced NSCLC using baseline and approximately 12-week CT	Prognostic study; 1171 discovery, 605 routine-practice tests, and 54 GARNET patients	Routine-practice test cohort and external trial cohort; RECIST and tumor-volume change comparators	Adjusted OS HR per 10-percentage-point increase in predicted 12-month survival: 0.74 (95% CI, 0.70–0.79) in routine-practice testing; 0.45 (0.32–0.65) in GARNET	Requires follow-up imaging; not a pretreatment selection model. Association with survival does not show benefit from score-guided treatment changes. Evidence stage: external prognostic validation.
[4]; issue 2026; full article	Comparison of pathology foundation models across morphological, biomarker, and prognostic tasks	Benchmark of 19 models; 13 cohorts; 6818 patients and 9528 slides across four cancer groups	Internal cross-validation and external cohorts; comparison between foundation models	CONCH had the highest overall performance; model performance varied by task and data setting	Pan-cancer benchmarking does not establish utility for an individual NSCLC decision. Evidence stage: comparative technical evaluation with external cohorts.
[5]; systematic review and meta-analysis	EGFR mutation prediction from whole-slide images	Sixteen studies in the review; four in the quantitative synthesis	Validation designs and reference assays inherited from the included studies	Pooled AUROC 0.756 (95% CI, 0.669–0.824); reported sensitivity 66.3% and specificity 68.1%	Pooled estimates do not define a safe threshold for withholding testing. Sensitivity/specificity CIs are not reproduced because the indexed abstract contains placeholders. Evidence type: secondary synthesis.
[6]; full article	Objective-response prediction from H&E slides in advanced NSCLC receiving ICI monotherapy	Multicenter cohort study; 614 development and 344 external-validation patients	External European cohort; PD-L1, with additional biomarker comparisons	External AUROC: Deep-IO 0.66 (95% CI, 0.60–0.72); PD-L1 0.67 (0.60–0.74); combined score 0.70 (0.63–0.76)	Response prediction within an ICI-treated population does not identify differential benefit versus another treatment. Evidence stage: external response-model validation.
[7]; full article	Response to PD-(L)1 blockade using imaging, pathology, and genomic features in advanced NSCLC	Retrospective multimodal cohort of 247 patients	Tenfold internal cross-validation; unimodal models and biomarkers. Additional cohorts evaluated individual modalities	Multimodal AUROC 0.80 (95% CI, 0.74–0.86); PD-L1 0.73 (0.65–0.81)	Additional unimodal evaluations are not external validation of the complete model. Modality availability and cohort selection affect transportability. Evidence stage: internally validated multimodal modeling.
[8]; conference abstract	Outcome stratification from pathology, with exploratory multimodal and treatment-selection applications	797 ICI-treated patients; 280 patients in external cohorts	External cohorts included Lung-MAP S1400I	S1400I survival-stratification HRs: OS 1.78, *p* = 0.016; PFS 2.76, *p* = 0.006; CIs not provided in the abstract	Reported HRs are not treatment-interaction estimates. Abstract-level reporting does not substantiate the claimed monotherapy-versus-chemoimmunotherapy selection rule. Evidence stage: preliminary external evaluation.
[9]; full article—contextual evidence	Biopsy strategy for peripheral nodules measuring 10–30 mm	Multicenter randomized noninferiority trial; reported analysis population 234, including five lost to follow-up	Navigational bronchoscopy versus transthoracic needle biopsy	Confirmed diagnostic accuracy: 94/119 (79.0%) versus 81/110 (73.6%); difference 5.4 percentage points (95% CI, −6.5 to 17.2)	Evaluates procedures rather than the incremental contribution of AI. Evidence type: contextual procedural trial.
[10]; full article—contextual evidence	Recurrence prediction after curative-intent treatment using tissue-informed ctDNA	Retrospective validation; LEMA 130 and LUCID 88 patients	Independent cohort evaluation; longitudinal ctDNA detection in relation to recurrence	Sensitivity/specificity: 61%/97% in LEMA and 64%/96% in LUCID; positivity defined by ≥1 positive sample ≥ 14 days after treatment	Longitudinal performance is not single-landmark performance; no estimate of incremental AI benefit or demonstrated utility of assay-guided treatment. Evidence type: contextual biomarker validation.

**Table 2:** Validation requirements according to the intended clinical use of an artificial intelligence system.

Intended Use	Acceptable Role	Required Validation	Essential Reporting
Measurement assistance	Output reviewed and correctable by a specialist	External technical and reader-workflow validation	Agreement, failure rate, time, subgroup performance
Triage or prioritisation	Changes order of review but not final diagnosis	Prospective workflow evaluation	Missed urgent cases, turnaround, queue effects, overrides
Reflex testing support	Recommends additional pathology or molecular testing	Multicenter prospective validation at target prevalence	Sensitivity, predictive values, calibration, tests added and yield
Rule-out or test replacement	Prevents a conventional diagnostic or molecular test	High-sensitivity external and interventional validation with safety follow-up	False negatives, delayed diagnoses, actionability missed, net benefit
Prognostic support	Informs counselling or follow-up intensity	Temporal and geographic validation against clinical baseline	Calibration, competing risks, clinical utility, subgroup safety
Treatment recommendation	Influences therapy choice	Comparative validation appropriate to the treatment decision, followed by prospective evaluation of model-guided care.	Differential benefit, patient outcomes, harms, override and accountability
Autonomous action	Acts without case-level human review	Regulatory approval plus rigorous prospective comparative evidence and surveillance	Fail-safe behaviour, drift, incidents, equity and version control

**Table 3:** Representative artificial intelligence applications across the NSCLC clinical pathway. Abbreviations: AI, artificial intelligence; CT, computed tomography; H&E, hematoxylin and eosin; NSCLC, non-small cell lung cancer; PET, positron emission tomography.

Clinical Phase	AI Task	Potential Value	Minimum Meaningful Endpoint	Principal Failure Risk
Risk assessment and screening	Eligibility refinement; future cancer-risk prediction	More personalized screening and intervals	Stage distribution, interval cancers, mortality or validated net benefit	Selection bias; poor transport across smoking and ancestry groups
CT interpretation	Nodule detection and segmentation	Higher sensitivity; reduced reading burden	Missed cancers, false positives per scan, workload and downstream procedures	Alert burden; scanner and protocol shift
Nodule management	Malignancy estimation	Better selection for surveillance, PET or biopsy	Unnecessary procedures avoided without delayed cancer	Miscalibration at local prevalence
Bronchoscopy	Route planning; tool-in-lesion and adequacy prediction	Higher diagnostic yield and better tissue allocation	Patient-level diagnostic yield and repeat-procedure rate	Optimization of navigation surrogate rather than diagnosis
Digital pathology	Tumour detection, subtype support and quantitative measurement	Consistency, quality control and time saving	Diagnostic error, turnaround time and pathologist-AI performance	Slide artefacts; weak performance in small biopsies and rare variants
Molecular triage	Prediction of actionable alteration from H&E or imaging	Faster prioritization; tissue stewardship	Testing completion, time to validated molecular results, and actionable alterations delayed or missed	False-negative denial of validated molecular testing
Prognosis	Recurrence or survival estimation	Risk-adapted follow-up and trial stratification	Calibration, decision utility and added value over stage	Interpreting outcome association as evidence supporting a treatment choice
Treatment selection	Estimation of differential benefit between treatment alternatives	Better allocation of systemic or local therapy	Prespecified treatment-effect interaction and outcomes of model-guided care	Confounding by indication; temporal leakage
Follow-up	Response, toxicity and recurrence detection	Earlier recognition and consistent measurement	Time to clinically useful action and outcome	Lead-time bias; treatment-related imaging change
Multidisciplinary care	Multimodal synthesis and evidence retrieval	More complete, timely and consistent decisions	Decision quality, concordance, safety and trial access	Missing-data shortcuts; hallucinated or outdated evidence

## Data Availability

No new data were created or analyzed in this study. Data sharing is not applicable to this article.

## Supplementary Materials

The following supporting information can be downloaded at: https://www.mdpi.com/article/10.3390/cancers18213415/s1. Table S1, Search sources, limits, and supplementary identification methods; Table S2, PubMed/MEDLINE search components and combinations; Table S3, Criteria used to prioritize evidence for narrative synthesis; Table S4, Operational terminology used in the review; Table S5, Study-specific methodological considerations and implications for interpretation.
