# Supplementary material for: Clinical Artificial Intelligence in Non-Small Cell Lung Cancer: From Early Detection and Computational Pathology to Precision Oncology
Source: Cancers (Basel). 2026 Oct 22;18(21):3415. doi: 10.3390/cancers18213415 (PMC13761698; doi:10.3390/cancers18213415)
Supplement: Supplementary file 1 [file cancers-18-03415-s001.zip › cancers-4588034-supplementary.pdf]

SUPPLEMENTARY MATERIAL

LITERATURE SEARCH AND REVIEW SCOPE

This supplementary file documents the search approach used to inform the state-of-the-art narrative review. The review was designed to identify clinically relevant and methodologically informative evidence rather than to provide an exhaustive systematic synthesis. Accordingly, study selection was purposive, no pooled effect estimate was calculated, and no formal risk-of-bias instrument was applied to every included publication.

PubMed/MEDLINE was searched from database inception to 31 August 2026. Searches were limited to English-language publications. Reference lists of relevant systematic reviews, methodological guidance, and pivotal original studies were examined to identify additional publications.

The study-level summaries distinguish direct evaluations of AI from contextual clinical evidence. Reported estimates were extracted from the cited sources without recalculation. The methodological observations in Supplementary Table S5 identify limitations affecting interpretation and do not constitute formal risk-of-bias ratings.

Item	Specification
**Review design**	State-of-the-art narrative review with purposive study selection; no claim of exhaustive retrieval.
**Publication types**	No publication-type filter was imposed. Full journal articles, conference abstracts, methodological guidance, and regulatory or trial-registry documents were distinguished in the synthesis.
**Scope**	NSCLC-focused clinical applications, with prediagnostic lung cancer evidence and general methodological studies included where relevant and identified as such.
**Supplementary identification**	Backward citation searching of relevant reviews, guidance, and primary studies.
**Selection and extraction**	Literature identification and selection: MC, SD. Extraction: MC, SD. Verification: LB.

**Synthesis**	Narrative synthesis of reported results; no new pooled estimates or reanalysis of primary-study data.
**Critical appraisal**	Qualitative consideration of study-specific limitations, informed by PROBAST+AI; no formal domain ratings or numerical quality scores.
**Coverage limitations**	Primarily PubMed/MEDLINE and English-language literature; technical publications and studies indexed elsewhere may be underrepresented.

Table S1. Search sources, limits, and supplementary identification methods.

REPRODUCIBLE PUBMED/MEDLINE STRATEGY

The strategy below documents the PubMed/MEDLINE search. S1–S18 are labels used in this supplement, not literal PubMed search-history identifiers. To execute the combinations, enter the component searches and substitute the corresponding history numbers assigned within the session. S10 is the broad search. S11–S18 are narrower combinations used to focus retrieval on individual domains; they do not extend retrieval beyond S10 when executed under the same conditions. Re-running the strategy later may produce different results because records and indexing can change.

Line	Search syntax
**S1**	("Carcinoma, Non-Small-Cell Lung"[Mesh] OR "Lung Neoplasms"[Mesh] OR NSCLC[tiab] OR "non-small cell lung cancer"[tiab] OR "non-small-cell lung cancer"[tiab] OR "lung cancer"[tiab] OR "lung neoplasm"[tiab] OR "lung neoplasms"[tiab])
**S2**	("Artificial Intelligence"[Mesh] OR "Machine Learning"[Mesh] OR "Deep Learning"[Mesh] OR "Neural Networks, Computer"[Mesh] OR "Natural Language Processing"[Mesh] OR "artificial intelligence"[tiab] OR "machine learning"[tiab] OR "deep learning"[tiab] OR "neural network"[tiab] OR "neural networks"[tiab] OR transformer[tiab] OR transformers[tiab] OR "foundation model"[tiab] OR "foundation models"[tiab] OR "large language model"[tiab] OR "large language models"[tiab] OR "computer vision"[tiab])
**S3**	(screening[tiab] OR "early detection"[tiab] OR "pulmonary nodule"[tiab] OR "pulmonary nodules"[tiab] OR "low-dose computed tomography"[tiab] OR LDCT[tiab])

**S4**	(radiology[tiab] OR imaging[tiab] OR radiomics[tiab] OR radiomic[tiab] OR "computed tomography"[tiab] OR CT[tiab] OR PET[tiab] OR "positron emission tomography"[tiab] OR staging[tiab] OR segmentation[tiab])
**S5**	(bronchoscopy[tiab] OR bronchoscopic[tiab] OR "robotic bronchoscopy"[tiab] OR "navigational bronchoscopy"[tiab] OR "tissue acquisition"[tiab] OR "diagnostic yield"[tiab])
**S6**	("digital pathology"[tiab] OR "computational pathology"[tiab] OR pathomics[tiab] OR pathomic[tiab] OR histopathology[tiab] OR histopathological[tiab] OR "whole-slide image"[tiab] OR "whole-slide images"[tiab] OR "whole slide imaging"[tiab])
**S7**	(genomics[tiab] OR genomic[tiab] OR molecular[tiab] OR mutation[tiab] OR mutations[tiab] OR "molecular profiling"[tiab] OR "molecular alteration"[tiab] OR "molecular alterations"[tiab] OR "liquid biopsy"[tiab] OR "circulating tumor DNA"[tiab] OR ctDNA[tiab] OR fragmentomics[tiab] OR methylation[tiab])
**S8**	(prognosis[tiab] OR prognostic[tiab] OR survival[tiab] OR recurrence[tiab] OR "treatment response"[tiab] OR predictive[tiab] OR immunotherapy[tiab] OR radiotherapy[tiab] OR "targeted therapy"[tiab])
**S9**	(multimodal[tiab] OR "multi-modal"[tiab] OR "decision support"[tiab] OR implementation[tiab] OR validation[tiab] OR calibration[tiab] OR workflow[tiab] OR governance[tiab] OR equity[tiab] OR surveillance[tiab] OR regulation[tiab])
**S10**	S1 AND S2 AND english[lang]
**S11**	S1 AND S2 AND S3 AND english[lang]
**S12**	S1 AND S2 AND S4 AND english[lang]
**S13**	S1 AND S2 AND S5 AND english[lang]
**S14**	S1 AND S2 AND S6 AND english[lang]
**S15**	S1 AND S2 AND S7 AND english[lang]
**S16**	S1 AND S2 AND S8 AND english[lang]
**S17**	S1 AND S2 AND S9 AND english[lang]
**S18**	S1 AND S2 AND (S3 OR S4 OR S5 OR S6 OR S7 OR S8 OR S9) AND english[lang]

Table S2. PubMed/MEDLINE search components and combinations. S labels refer to the rows below; actual search-history identifiers depend on the PubMed session. The reported search date was 31 August 2026.

STUDY PRIORITIZATION FRAMEWORK

Because the review was narrative, the following criteria were used to prioritize evidence rather than as formal inclusion and exclusion criteria. A study could be retained despite not meeting every criterion when it was historically seminal or addressed a clinically important evidence gap.

Domain	Priority features	Lower-priority features
**Clinical relevance**	A defined intended use linked to a real clinical decision, workflow, or patient outcome.	Technical classification task without a specified clinical action or comparator.
**Validation**	Evaluation in data separated from model development and tuning, with temporal, institutional, geographical, or other differences relevant to the intended use; prospective workflow evaluation where appropriate.	Evaluation limited to an internal random split, reuse of evaluation data for tuning, or unclear patient-level separation. Multicenter data collection alone does not establish external validation.
**Comparator**	Comparison with current standard care, validated clinical models, or clinician performance with and without AI.	Comparison with chance, an obsolete baseline, or unaided clinicians in an artificial reader setting.
**Outcomes**	Outcomes matched to the intended use, with uncertainty estimates and, where applicable, calibration, prespecified operating thresholds, workflow effects, harms, or patient outcomes.	AUROC or accuracy reported without calibration, threshold-specific performance, or downstream consequences.
**Population and data**	Representative case mix, explicit specimen and acquisition provenance,	Enriched case-control design, exclusion of technically failed cases, or poorly described sampling.

	and clinically meaningful subgroup analysis.	
**Transparency**	Clear reference standard, preprocessing, data partitioning, missing-data handling, uncertainty, and failure policy.	Insufficient information to assess leakage, spectrum bias, missingness, or model failure.
**Treatment claims**	Direct comparison of clinically relevant treatments, with a prespecified treatment-by-model interaction or an appropriate causal analysis; prospective evaluation when claiming benefit from model-guided treatment.	Response or survival prediction within a single treatment cohort interpreted as evidence that the model identifies the preferable treatment.

Table S3. Criteria used to prioritize evidence for narrative synthesis.

OPERATIONAL DEFINITIONS

Term	Definition used in this review
**Artificial intelligence**	Umbrella term for computational systems performing tasks that ordinarily require human cognitive interpretation or decision support.
**Machine learning**	Models that learn relationships from data rather than relying solely on explicitly programmed rules.
**Deep learning**	Machine-learning methods based on multilayer neural networks, including convolutional networks and transformers.
**Radiomics**	Extraction and analysis of predefined quantitative features from medical images.
**Deep radiomics**	Learning of imaging representations directly from image data rather than relying only on predefined handcrafted features.

**Computational pathology**	Application of computational image analysis and related models to digitized tissue or cytology for diagnostic, quantitative, prognostic, or molecular inference tasks.
**Pathomics**	Quantitative characterization of morphological, cellular, and spatial features from digitized pathological material.
**Multimodal model**	A model that integrates two or more data modalities, such as imaging, pathology, molecular measurements, clinical variables, or text.
**Foundation model**	A model pretrained on large and heterogeneous datasets that can be adapted to multiple downstream tasks.
**External validation**	Evaluation using data not involved in development, model selection, or tuning, with a separation relevant to the intended application, such as institution, geography, or time. Multicenter development alone does not constitute external validation.
**Clinical utility**	Evidence that using a model improves decisions, workflow, resource use, harms, or patient outcomes compared with an appropriate alternative.
**Silent evaluation**	Prospective evaluation using live workflow data in which model output does not influence patient care.
**Prognostic model**	A model estimating the probability of a future outcome in a defined population and clinical setting.
**Treatment-response prediction**	Estimation of the probability of response to a treatment; this does not necessarily identify whether that treatment is preferable to an alternative.
**Treatment-effect prediction**	Estimation of how outcomes differ between treatment alternatives according to patient or model characteristics, requiring a suitable comparative design.
**Clinical implementation**	Use of a system within an actual care process, with defined users, actions, responsibilities, and monitoring arrangements.
**Contextual evidence**	Evidence relevant to the clinical task or evaluation standard that does not directly estimate the performance or incremental contribution of AI.

Table S4. Operational terminology used in the review.

Study	Methodological detail	Implications for interpretation
**Sybil**	Evaluation included a held-out NLST dataset and external MGH and CGMH cohorts. Examination counts differ from patient counts because some patients contributed multiple scans.	Held-out testing within NLST and external validation provide different evidence of generalizability. Risk discrimination alone does not establish the benefit of changing screening eligibility or intervals.
**LungIMPACT**	AI was available in both arms; worklist prioritization was randomized by day. The intervention modified one step of an existing diagnostic pathway.	The trial estimates the effect of AI-based prioritization, rather than AI availability. Faster reporting did not demonstrate faster downstream diagnosis; nonsignificant differences do not establish equivalence.
**Serial-CT model**	The model uses baseline and approximately 12-week CT scans. The external GARNET cohort included 54 patients, separate from the discovery and routine-practice test cohorts.	The score provides on-treatment prognostic information. Applicability depends on follow-up imaging availability, the survival-analysis time origin, and the handling of patients who deteriorate or die before reassessment.
**Foundation-model benchmark**	Evaluation covered multiple cancer types and downstream tasks. The authors described external datasets and selected CPTAC collections without TCGA patient overlap.	Independence requires consideration of both foundation-model pretraining and downstream model development. Performance across heterogeneous benchmark tasks does not establish clinical utility for a specific NSCLC application.
**EGFR meta-analysis**	Sixteen studies were included in the systematic review, but only four contributed to the meta-analysis. The indexed abstract contains incomplete confidence	The pooled estimates represent a subset of the reviewed studies. Missing uncertainty estimates cannot be inferred from the available summary. Pooled discrimination does not establish a safe threshold for withholding molecular testing.

	intervals for sensitivity and specificity.	
**Deep-IO**	Development and external validation involved patients receiving ICI monotherapy. Performance was assessed against response and survival outcomes, with biomarker comparisons.	Response prediction within an ICI-treated population does not establish differential benefit between treatment alternatives. Similar discrimination to PD-L1 does not demonstrate formal equivalence.
**Vanguri multimodal model**	The complete multimodal model was evaluated using tenfold cross-validation. Additional cohorts of 50 and 52 patients evaluated radiological and pathological features, respectively. The study also examined modality contributions.	The additional cohorts do not constitute external validation of the complete multimodal model. Analyses of modality contributions do not establish transportability to a new population with different patterns of data availability.
**Path-IO**	Findings were reported in a conference abstract. External evaluation included S1400I, which compared nivolumab with nivolumab plus ipilimumab. The abstract does not provide a treatment-by-score interaction estimate with uncertainty.	Abstract-level reporting limits assessment of treatment-effect modification. Survival-stratification hazard ratios are not treatment-interaction estimates. S1400I cannot establish a selection rule between ICI monotherapy and chemoimmunotherapy.
**VERITAS**	The trial compared biopsy procedures. Diagnostic accuracy required a specific diagnosis confirmed through 12 months of follow-up.	Diagnostic accuracy differs from navigation success and from yields defined using other criteria. Comparisons with unrelated procedural series require attention to population, platform, and follow-up differences. The trial does not isolate the contribution of AI.

**LEMA/LUCID**	Reported sensitivity and specificity concern longitudinal ctDNA detection from ≥14 days after treatment. The 88% NPV discussed in relation to de-escalation concerned stage II–III patients with detectable ctDNA before treatment but no detection afterward.	Longitudinal performance cannot be transferred to a single postoperative landmark assessment. The NPV statement does not concern stage I disease. Recurrence-risk stratification does not establish the benefit of assay-guided treatment changes or the incremental contribution of AI.

Table S5. Study-specific methodological considerations and implications for interpretation. These observations provide a qualitative appraisal of the cited studies, not formal PROBAST+AI ratings. Incomplete reporting does not establish that a procedure or analysis was not performed. No primary-study analyses were repeated. Abbreviations: AI, artificial intelligence; CGMH, Chang Gung Memorial Hospital; CPTAC, Clinical Proteomic Tumor Analysis Consortium; CT, computed tomography; ctDNA, circulating tumor DNA; ICI, immune checkpoint inhibitor; MGH, Massachusetts General Hospital; NLST, National Lung Screening Trial; NPV, negative predictive value; NSCLC, non-small cell lung cancer; PD-L1, programmed death ligand 1; TCGA, The Cancer Genome Atlas.
